# Molecular detection of pathogenic bacteria in the colonic biopsies from patients with Ulcerative Colitis

**DOI:** 10.4314/ahs.v22i1.70

**Published:** 2022-03

**Authors:** Thanaa El A Helal, Hoda E El Abdel Wahab, Sally M Saber, Waleed H Abdelaaty, Mohamed M Eltabbakh, Ahmed M Aref, Mohamed H Dawood

**Affiliations:** 1 Department of Pathology, Faculty of Medicine, Ain Shams University, Cairo, Egypt; 2 Department of Clinical Pathology, Faculty of Medicine, Ain Shams University, Cairo, Egypt; 3 Department of Tropical Medicine, Faculty of Medicine, Ain Shams University, Cairo, Egypt; 4 Faculty of Biotechnology, October University for Modern Sciences and Arts (MSA), Giza, Egypt

**Keywords:** Ulcerative colitis, Colonic biopsies, *Clostridium difficile*, *H. pylori*, *E. coli*

## Abstract

**Background/Aim:**

Ulcerative Colitis (UC) is an inflammatory bowel disease which is common in many areas of the world including Egypt. A lot of controversy regarding the pathogenesis of UC exist. The current study is an attempt to detect some pathogenic bacteria in UC patients.

**Materials and methods:**

Endoscopic colonic biopsies obtained from 40 patients with ulcerative colitis and 20 controls were analyzed by means of real-time PCR technique for the presence of *Clostridium difficile, Helicobacter Pylori* (*H. pylori*) and pathogenic *Escherichia Coli* (*E. coli)* which are positive for KPC and/or OXA-48.

**Results:**

All patients and control samples were negative for *Clostridium difficile*. Three of the 40 patient samples (7.5%) and none of the 20 controls were positive for *H. pylori* with no significant difference between the two groups. KPC-positive *E. coli* were detected in 11 of the 40 patients (27.5%) and in none of the controls with a significant difference between the two groups (P=0.01). All patients and control samples were negative for OXA-48 positive E. coli.

**Conclusion:**

Although this study does not support the claim that *Clostridium difficile* and/or *H. pylori* have a role in UC, it greatly suggests that pathogenic *E. coli* may be involved in one way or another in the course of UC.

## Introduction

Ulcerative Colitis (UC) is an inflammatory bowel disease which occurs with different frequencies around the world with the highest incidence in Canada, United States, United Kingdom and Sweden 1, 2. Although no accurate data about the exact prevalence of UC in the Middle East, some studies pointed out that the incidence of UC is increasing in this area of the world due to changes in the lifestyle 3, 4.

The pathogenesis of UC has been a subject of much controversy. Several authors believed that UC may be induced by pathogenic bacteria. *Shigella, Salmonella* and Yersinia have been suggested as possible cause of UC 5. More recent studies have argued that pathogenic *Escherichia Coli* (*E. coli*) belonging to the B2 and D subgroups play an important role in the pathogenesis of UC 6, 7. Moreover, other investigators have shown that UC patients have a high risk of Clostridium difficile infection when compared with healthy individuals^8^, ^9^. Also *Helicobacter Pylori* (*H. pylori*) was found colonizing to the gastric mucosa 10. The relationship between *H. pylori* and UC is controversial. Some studies concluded that *H. pylori* has a protective role against UC 11–13. Contradictory results have been reported by other investigators who claimed that H. pylori may have a causative role in UC 14, 15.

In the current study we investigated the presence of Clostridium difficile, *H. pylori* and pathogenic *E. coli* in the colonic tissue specimens from patients with UC.

## Materials and Methods

### Study Subjects and specimen collection

This is a retrospective study which was performed on formalin fixed, paraffin-embedded (FFPE) colonic tissue specimens obtained by endoscopy from 40 patients with ulcerative colitis (UC). They were 21 females and 19 males. Their median age was 40 years. Twenty two specimens obtained from rectum or rectosigmoid and 18 were obtained from left colon.

Diagnosis of UC was based on the clinical picture, endoscopic and histologic findings 16. It is worth mentioning that 4 of 40 UC patients (10%) had helicobacter gastritis as proved by gastric biopsy done prior to the study. The disease activity was assessed histologically according to Gupta et al 17. All patients received no antibiotics for at least 2 months before taking the biopsy. Twenty control specimens were obtained from age-matched individuals who underwent endoscopy because of abdominal pain or discomfort. They showed normal endoscopic and histologic findings.

This work was approved by the institutional review board of Ain Shams faculty of Medicine which waived the requirement for informed consent because it was a retrospective study and the cases were analyzed in an anonymous way.

Real-time PCR procedure: Genomic DNA extraction from the 60 formation-fixed paraffin embedded colonic tissue specimens (40 UC patients and 20 controls) was performed using the DNA purification Kit (QIA amp DNA FFPE kit, Qiagen, Germany). The steps were done following the manufacturer protocol.

DNA amplification and detection was performed using Quanti-tech SYBR Green PCK Kit (Qiagen, Germany). A PCR reaction was done to amplify the primers as shown in (Table 1). Ten microliter of the template DNA was added to the reaction mixture following the instructions of the manufactures. PCR was performed using Roter Gene 5 plex machine (Qiagen, Germany). The amplification protocol included initial denaturation step at 94°C for 15 min, followed by 45 cycles which consisted of annealing at 55°C for 30 seconds and extension at 72°C for 30 seconds 18.

**Table 1:** Primers used in this study

Pathogen	Gene		Primer	Reference number
***Clostridium*** ***difficile***	Tyi	Forward Reverse	AAGAAGCTACTAAGGGTACAAA CATAATATTGGGTCTATTCCTAC	35
***H. pylori***	VAC A	Forward Reverse	ATGGAAATACAACAAACACAC CTGCTTGAATGCGCCAAAC	36
***E coli***	16S rDNA	Forward Reverse	CATGCCGCGTGTATGAAGAA CGGGTAACGTCAATGAGCAAA	37
	**Bla (KPC)**	Forward Reverse	TCGCTAAACTCGAACAGG TTACTGCCCGTTCACGCCCAATCC	18
	**Bla (oxa-48)**	Forward Reverse	TGTTTTTGGTGGCATCGAT GTAAMRATGCTTGGTTCGC	18

**KPC:** Klebsiella pneumoniae Carbapenemase Producing gene.

**Bla OXA-48:** Beta lactamase producing gene (oxacillinase).

An additional test was performed to detect Carbapenem resistant E. coli (MDR) using the KPC and OXA-48 resistant gene primers according to Monterio et al. 2012 18 as shown in (Table 1). The same conditions and reagents quantities were applied.

The results were interpreted by melting curve analysis. It is an assessment of the dissociation characteristics of double-stranded DNA during heating. As the temperature is raised, the double strand begins to dissociate leading to a rise in the absorbance intensity. The temperature at which 50% of DNA denaturated is known as melting point 19.

### Statistical analysis

Descriptive data were presented as count and percentages. Quantitative variables were given as median. The statistical analysis was performed using Fisher's exact test and the statistical software SPSS, version 16, A value of P ≤ 0.05 was considered significant.

## Results

Endoscopic examination showed that 18 of the 40 UC patients (45%) had Pancolitis, 15 patients (37.5%) had left sided colitis and 7 patients (17.5%) had proctitis.

They were classified into 12(30%) having active disease and 28 (70%) having quiescent disease. Inflammation was marked in 15 patients (including the 12 with active disease), moderate in 9 and mild in 16 cases. Low grade dysplasia was detected in 5 cases (12.5%). No case had high grade dysplasia.

**Figure (1):**
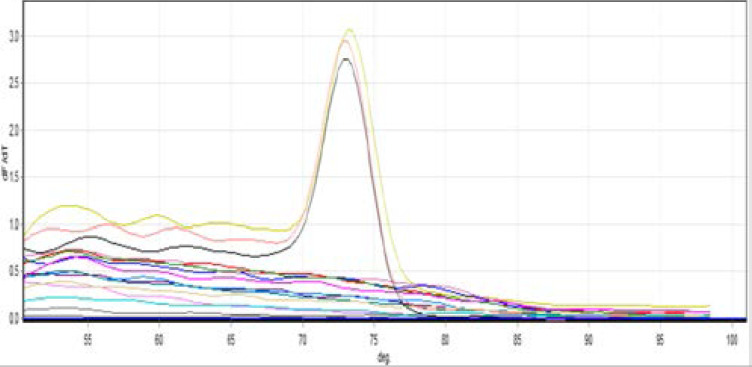
Melting curve shows 3 positive *H. pylori* samples

All tissue specimens from patients and controls were negative for Clostridium difficile. *H. pylori* were identified in 3 of the 40 UC specimens (7.5%). They were absent in all 20 control tissue specimens (Figure 1). The difference was not statistically significant (P>0.05). No significant association could be detected between *H. pylori* infection and the patient age, gender or disease activity (P>0.05) (Table 2).

**Table 2:** Relationship between *H. pylori* and patients' samples

Patient data	*H. pylori* +ve	*H. pylori* -ve	Fisher exact test value	P-value
**Age (years)** > 40 < 40	2 1	20 17	1	NS
**Gender** Male Female	2 1	17 20	0.596	NS
**Activity** **Active** **Inactive**	2 1	10 27	0.209	NS

NS: Not significant

**Figure (2):**
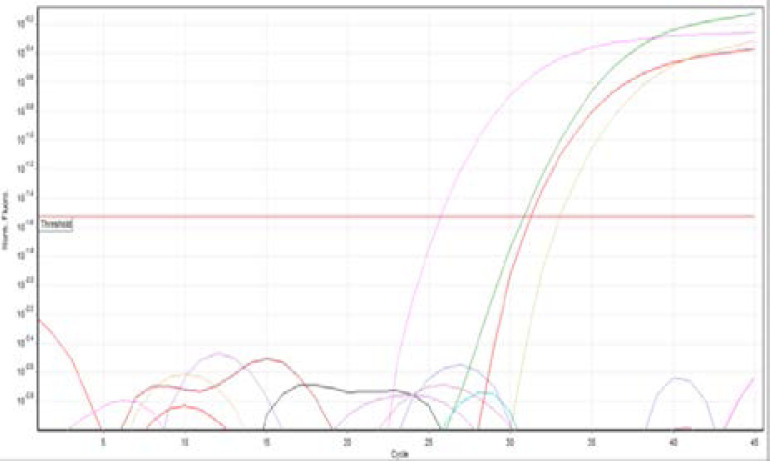
positive *E. coli samples* (indicated by the elevated peaks)

**Figure (3):**
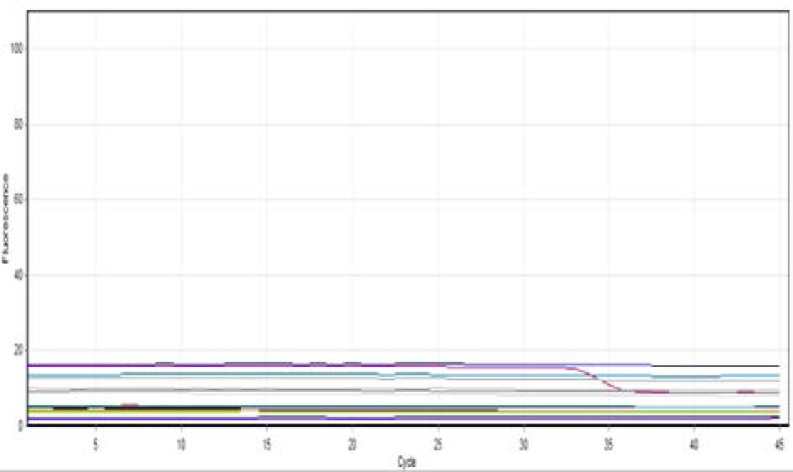
shows all-negative BlaOXA48 *E. coli* samples

**Figure (4):**
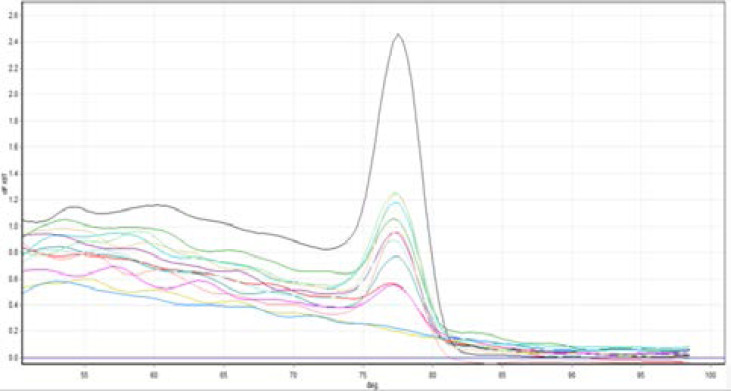
KPC positive samples with one negative KPC control sample

Commensal *E. coli* were detected in all 60 tissue specimens included in the study (40 patients and 20 controls) as shown in (Figure 2). Also, all samples were negative for Bla OXA48 positive E. coli as shown in (Figure 3), However, 11 of the 40 UC tissue specimens were KPC positive (27.5%) as compared to the controls which all KPC negative (Figure 4). The difference was statistically significant (Fisher exact test value 0.011, p=0.01) as shown in (Table 3).

**Table 3:** Comparing patients and controls regarding KPC

	Patients	controls	Fisher exact test value	P-value
**KPC +Ve**	11	0	0.001	0.01 Sig
**KPC -Ve**	29	20		
**Total**	40	20		

**Sig**: significant

**NS**: Not significant

There was no significant association between KPC positively and patient age, gender or disease activity (P>0.05) (Table 4).

**Table 4:** Relationship between pathogenic *E. coli* KPC and patients data

Patient data	*E. coli* +ve	*E. coli* -ve	Fisher exact test value	P-value
**Age (years)** > 40 > 40	7 4	15 14	0.724	NS
**Gender** Male Female	4 7	15 14	0.488	NS
**Activity** **Active** **Inactive**	3 8	9 20	1.00	NS

**NS**: Not significant

## Discussion

Ulcerative Colitis (UC) is an inflammatory disease which affects the colon and rectum. Many factors are thought to contribute to the pathogenesis of UC including genetic, host immune system disorders, intestinal bacteria, and environmental factors. However, studies on the role of intestinal bacteria in the pathogenesis of UC have been inconclusive 1, 2.

In the current study we investigated the presence of Clostridium difficile, H. pylori and pathogenic E. coli in the colonic tissue from patients with UC, using real-time PCR technique.

We could not detect Clostridium difficile in all specimens from patients and controls. This results contradicts that of Lin et al. and Shoaei et al. 20, 21 who detected Clostridium difficile in 17.6% and 29.4% of UC patients respectively. In fact, this contradiction was expected since the previous two studies were performed on fecal samples while our study was performed on colonic tissue. It is well known that stool analysis for bacteria may give much higher false positive results than tissue analysis since most bacteria live within the intestinal lumen and don't enter the mucosa 22. That is why, Lin et al concluded that microbial analysis of colonic tissue samples may give more solid data than stool analysis 24, 25. Amre et al. reported that the low prevalence of H. pylori infection in patients with inflammatory bowel disease may explain the role of the hygienic hypothesis in the development of this disease. The authors speculated that inadequate microbial stimulation of the gut-associated lymphoid tissue leads to maturation of the mucosal immunity 26. This speculation is supported by the study of Koloski et al. who reported that a clean environment decreases the incidence of common infections as *H. pylori*. This leads to autoimmunity and increased susceptibility to certain autoimmune diseases as UC 27.

The most important organism investigated in this study was E. coli. We detected that 27.5% of our UC patients had KPC positive *E coli* (MDR resistant) which are most likely pathogenic. It is interesting to note that detection of pathogenic E. coli in the colonic tissue of UC patients has been also reported by Kotlowski et al. who identified E. coli belonging to the B2 group which are known to be pathogenic 28. Moreover, *E. coli* strains positive for pathogenecity factors ompA, afae and USP were detected in UC patients 7. In a recent study by Meheissen et al. they identified pathogenic E. coli strainsin 25% (15/60) of inflammatory bowel disease (IBD) cases and in none of the controls 29.

Other studies were performed on the commensally *E. coli* and found that their number was significantly higher than the controls and concluded that E coli may play a role in the pathogenesis of UC 30–32. More recently, Pilarczyk-Zurek et al. pointed out that *E. coli* have a dual role in the course of UC. One role is initiation of inflammation and the other is that *E. coli* may help induction of remission of UC^33^.

The limitation of the current study is the small number patients included and using FFPE tissue samples. However, it has the advantage of using colonic tissue, rather than stool sample, thus giving more reliable data. Also, we used real-time PCR technique which is an accurate technique to reassure the amplicon by monitoring the accumulation of specific product during each cycle and discriminating it from outliers through melting curve analysis. Moreover, amplification and detection was performed in the same tube (close tube system). This decreases the possibility of contamination and false positive result 34. At the same time, the primers used in the study were species specific. So, they only anneal to the templates from one species, thus increasing the accuracy of the results 35–37.

## Conclusion

The present study suggests that Clostridium difficile and *H. pylori* seem to have no role in UC. More importantly, the detection of pathogenic *E. coli* in 27.5% of our UC patients suggests that these bacteria may be involved in the process of inflammation in one way or another. This point of research is still in its beginning, and a lot of future studies are recommended to clarify the relationship between *E. coli* and UC.

## Approval of final manuscript

All authors.

## References

[1] Ng SC, Shi HY, Hamidi N, Underwood FE, Tang W, Benchimol EI (2018). Worldwide incidence and prevalence of inflammatory bowel disease in the 21st century: a systematic review of population-based studies. Lancet.

[2] Kaplan GG, Ng SC (2016). Globalisation of inflammatory bowel disease: perspectives from the evolution of inflammatory bowel disease in the UK and China. Lancet Gastroenterol Hepatol.

[3] Abdul-Baki H, ElHajj I, El-Zahabi LM, Azar C, Aoun E, Zantout H (2007). Clinical epidemiology of inflammatory bowel disease in Lebanon. Inflamm Bowel Dis.

[4] Ouakaa-Kchaou A, Gargouri D, Bibani N, Elloumi H, Kochlef A, Kharrat J (2013). Epidemiological evolution of epidemiology of the inflammatory bowel diseases in a hospital of Tunis. Tunis Med.

[5] Rhodes JM (1996). Unifying hypothesis for inflammatory bowel disease and associated colon cancer: sticking the pieces together with sugar. Lancet.

[6] Schippa S, Conte MP, Borrelli O, Iebba V, Aleandri M, Seganti L (2009). Dominant genotypes in mucosa-associated Escherichia Coli strains from pediatric patients with inflammatory bowel disease. Inflamm Bowel Dis.

[7] Sepehri S, Khafipour E, Bernstein CN, Coombes BK, Pilar AV, Karmali M (2011). Characterization of Escherichia Coli isolated from gut biopsies of newly diagnosed patients with inflammatory bowel disease. Inflamm Bowel Dis.

[8] Nguyen GC, Kaplan GG, Harris ML, Brant SR (2008). A national survey of the prevalence and impact of Clostridium difficile infection among hospitalized inflammatory bowel disease patients. Am J Gastroenterol.

[9] Berg AM, Kelly CP, Farraye FA (2013). Clostridium difficile infection in the inflammatory bowel disease patient. Inflamm Bowel Dis.

[10] Dunn BE, Cohen H, Blaser MJ (1997). Helicobacter pylori. Clin Microbiol Rev.

[11] Jin X, Chen YP, Chen SH, Xiang Z (2013). Association between Helicobacter Pylori infection and ulcerative colitis--a case control study from China. Int J Med Sci.

[12] Sonnenberg A, Genta RM (2012). Low prevalence of Helicobacter Pylori infection among patients with inflammatory bowel disease. Aliment Pharmacol Ther.

[13] Wu XW, Ji HZ, Yang MF, Wu L, Wang FY (2015). Helicobacter Pylori infection and inflammatory bowel disease in Asians: a meta-analysis. World J Gastroenterol.

[14] Streutker CJ, Bernstein CN, Chan VL, Riddell RH, Croitoru K (2004). Detection of species-specific helicobacter ribosomal DNA in intestinal biopsy samples from a population-based cohort of patients with ulcerative colitis. J Clin Microbiol.

[15] Mansour L, El-Kalla F, Kobtan A, Abd-Elsalam S, Yousef M, Soliman S (2018). Helicobacter Pylori may be an initiating factor in newly diagnosed ulcerative colitis patients: A pilot study. World J Clin Cases.

[16] Ibd Working Group of the European Society for Paediatric Gastroenterology H and Nutrition (2005). Inflammatory bowel disease in children and adolescents: recommendations for diagnosis--the Porto criteria. J Pediatr Gastroenterol Nutr.

[17] Gupta RB, Harpaz N, Itzkowitz S, Hossain S, Matula S, Kornbluth A (2007). Histologic inflammation is a risk factor for progression to colorectal neoplasia in ulcerative colitis: a cohort study. Gastroenterology.

[18] Monteiro J, Widen RH, Pignatari AC, Kubasek C, Silbert S (2012). Rapid detection of carbapenemase genes by multiplex real-time PCR. J Antimicrob Chemother.

[19] Muldrew KL (2009). Molecular diagnostics of infectious diseases. Curr Opin Pediatr.

[20] Lin WC, Chang CW, Chen MJ, Chu CH, Shih SC, Hsu TC (2017). Challenges in the diagnosis of ulcerative colitis with concomitant bacterial infections and chronic infectious colitis. PLoS One.

[21] Shoaei P, Shojaei H, Jalali M, Khorvash F, Hosseini SM, Ataei B (2019). Clostridium difficile isolated from faecal samples in patients with ulcerative colitis. BMC Infect Dis.

[22] Ott SJ, Musfeldt M, Ullmann U, Hampe J, Schreiber S (2004). Quantification of intestinal bacterial populations by real-time PCR with a universal primer set and minor groove binder probes: a global approach to the enteric flora. J Clin Microbiol.

[23] Joshi NM, Marks IH, Crowson R, Ball D, Rampton DS (2017). Incidence and Outcome of Clostridium difficile Infection in Hospitalized Patients with Inflammatory Bowel Disease in the UK. J Crohns Colitis.

[24] Thomson JM, Hansen R, Berry SH, Hope ME, Murray GI, Mukhopadhya I (2011). Enterohepatic helicobacter in ulcerative colitis: potential pathogenic entities?. PLoS One.

[25] Mukhopadhya I, Thomson JM, Hansen R, Berry SH, El-Omar EM, Hold GL (2011). Detection of Campylobacter concisus and other Campylobacter species in colonic biopsies from adults with ulcerative colitis. PLoS One.

[26] Amre DK, Lambrette P, Law L, Krupoves A, Chotard V, Costea F (2006). Investigating the hygiene hypothesis as a risk factor in pediatric onset Crohn's disease: a case-control study. Am J Gastroenterol.

[27] Koloski NA, Bret L, Radford-Smith G (2008). Hygiene hypothesis in inflammatory bowel disease: a critical review of the literature. World J Gastroenterol.

[28] Kotlowski R, Bernstein CN, Sepehri S, Krause DO (2007). High prevalence of Escherichia Coli belonging to the B2+D phylogenetic group in inflammatory bowel disease. Gut.

[29] Meheissen M, Header D, Abdelaty K (2019). Phylogenetic and pathotype analysis of Escherichia Coli stool isolates from Egyptian patients with inflammatory bowel disease. Germs.

[30] Mylonaki M, Rayment NB, Rampton DS, Hudspith BN, Brostoff J (2005). Molecular characterization of rectal mucosa-associated bacterial flora in inflammatory bowel disease. Inflamm Bowel Dis.

[31] Kleessen B, Kroesen AJ, Buhr HJ, Blaut M (2002). Mucosal and invading bacteria in patients with inflammatory bowel disease compared with controls. Scand J Gastroenterol.

[32] Pilarczyk-Zurek M, Chmielarczyk A, Gosiewski T, Tomusiak A, Adamski P, Zwolinska-Wcislo M (2013). Possible role of Escherichia Coli in propagation and perpetuation of chronic inflammation in ulcerative colitis. BMC Gastroenterol.

[33] Pilarczyk-Zurek M, Strus M, Adamski P, Heczko PB (2016). The dual role of Escherichia Coli in the course of ulcerative colitis. BMC Gastroenterol.

[34] Soheili Z aSS (2005). real time PCR. Hepatitis Monthly.

[35] Blake LW AP, Ramsubhag A, Blake RK (2015). Molecular and Susceptibility Analysis of Toxigenic Clostridium difficile Obtained from Adult Patients Suspected of CDI in Trinidad. Open J Med Microbiol.

[36] Bagheri N, Azadegan-Dehkordi F, Shirzad M, Zamanzad B, Rahimian G, Taghikhani A (2015). Mucosal interleukin-21 mRNA expression level is high in patients with Helicobacter Pylori and is associated with the severity of gastritis. Cent Eur J Immunol.

[37] Huijsdens XW, Linskens RK, Mak M, Meuwissen SG, Vandenbroucke-Grauls CM, Savelkoul PH (2002). Quantification of bacteria adherent to gastrointestinal mucosa by real-time PCR. J Clin Microbiol.

